# Retraction: [Pt(*O,O’*-acac)(γ-acac)(DMS)] Alters SH-SY5Y Cell Migration and Invasion by the Inhibition of Na^+^/H^+^ Exchanger Isoform 1 Occurring through a PKC-ε/ERK/mTOR Pathway

**DOI:** 10.1371/journal.pone.0353999

**Published:** 2026-07-20

**Authors:** The PLOS One Editors

After this article [1,2] was published, concerns were raised regarding Fig 1-6. Specifically,

- The following panels within Figs 1 and 4 appear to partially overlap with one another:
  - The upper left and lower right panels of Fig 1B, when rotated 180^o^.
  - The lower left and upper right panels of Fig 1B.
  - Lane 2 in the MMP-9 and MMP-2 panels of Fig 4D.
- There appear to be similarities within the following panels of Figs 3 and 6:
  - The backgrounds of lanes 1 and 3 of the p-ERK1/2 panel of Fig 3B.
  - Lanes 1 and 4 of the β-actin panel of Fig 3B.
  - Lanes 3 and 4 of the ɑ-Na^+^/K^+^ATPasi panel of Fig 6B.
- The following panels in Figs 3 and 5 appear to contain vertical discontinuities;
  - The p-6S [Pt(acac)_2_(DMS)] (0.50μM) panel of Fig 3B, between lanes 3–4.
  - The p-S6 [Pt(acac)_2_(DMS)] panel of Fig 5A, between lanes 3–4.
- The following panels in Figs 2 and 4 appear to overlap with later articles published by the same research group:
  - Lanes 1–4 of the [Pt(acac)_2_(DMS)] (0.5μM) ERK 1/2 panel of Fig 2D and the upper ERK 1/2 panel of Fig 6A in [3].
  - Lanes 1–4 of the β-actin panel of Fig 4C and the β-actin panel of Fig 4A in [4], when flipped horizontally.
  - Lanes 1–4 of the p38MAPK panel of Fig 4C and the β-actin panel of Fig 6D in [4], when flipped horizontally.

The corresponding author stated that any perceived similarities between images are possibly the result of artefacts arising during manual development of chemiluminescent films.

In light of the above concerns that question the reliability and integrity of the reported results and conclusions, the *PLOS One* Editors retract this article.

AM, FPF, and SM did not agree with retraction. CV, NC, LGC, and SADP either did not respond directly or could not be reached.
